# Bovine milk-derived cells express transcriptome markers of pluripotency and secrete bioactive factors with regenerative and antimicrobial activity

**DOI:** 10.1038/s41598-023-39833-9

**Published:** 2023-08-03

**Authors:** Nikola Danev, Rebecca M. Harman, Leane Oliveira, Lucas Huntimer, Gerlinde R. Van de Walle

**Affiliations:** 1 Baker Institute for Animal Health, College of Veterinary Medicine, Cornell University, 235 Hungerford Hill Road, Ithaca, NY 14853 USA; 2 Elanco Animal Health, 2500 Innovation Way, Indianapolis, IN 46241 USA

**Keywords:** Cell biology, Stem cells

## Abstract

The bovine mammary stem/progenitor cell secretome stimulates regeneration in vitro and contains proteins associated with antimicrobial defense. This has led to the exploration of the secretome as a biologic treatment for mastitis, a costly inflammation of the udder commonly caused by bacteria. This study reports on a population of bovine mammary stem/progenitor cells isolated non-invasively from milk (MiDCs). MiDCs were characterized by immunophenotyping, mammosphere formation assays, and single cell RNA sequencing. They displayed epithelial morphology, exhibited markers of mammary stem/progenitor cells, and formed mammospheres, like mammary gland tissue-isolated stem/progenitor cells. Single cell RNA sequencing revealed two sub-populations of MiDCs: epithelial cells and macrophages. Functionally, the MiDC secretome increased fibroblast migration, promoted angiogenesis of endothelial cells, and inhibited the growth of mastitis-associated bacteria, including antibiotic-resistant strains, in vitro. These qualities of MiDCs render them a source of stem cells and stem cell products that may be used to treat diseases affecting the dairy industry, including mastitis.

## Introduction

The United States dairy industry constitutes 3.5% of the Gross Domestic Product, for an economic impact of $752.93 billion^1^. The most common infectious disease in dairy cows is mastitis, an inflammation of the udder, typically associated with bacterial infection^2,3^. Mastitis is usually treated with conventional antibiotics^4^. It is an economically costly disease due to the price of treatment and the fact that milk from cows either diagnosed with mastitis or being treated with conventional antibiotics cannot be sold for human consumption, and is typically discarded^5–7^. In addition, mastitis can lead to a permanent decrease in milk yield due to the irreperable damage of the parenchymal epithelial tissue in the udder, which is not restored by antibiotic treatment^8^. Finally, certain mastitis-causing bacteria have developed antimicrobial resistance rendering current antibiotic treatment regimens ineffective^9^. The use of conventional antibiotics in the treatment of food-producing animals is associated with an increase in the prevalence of resistant bacteria in both humans and animals^10^, necessitating the use of next-generation antibiotics^11^. The cost of developing one new, next-generation antibiotic exceeds $1.5 billion, a cost that created bottlenecks in availability and development^12^, and many next-generation antibiotics are restricted for use in humans^13^, as outlined in the World Health Organization List of Critically Important Antimicrobials for Human Medicine^14^. These limitations of using conventional antibiotics have led to the need for alternative mastitis treatments, ideally therapies that can restore udder damage to achieve pre-mastitis milk yields.

Stem cells are long-lived clonogenic cells capable of both self-renewal and multilineage differentiation that can be isolated from embryonic or adult tissues^15,16^. Stem cells actively secrete bioactive factors, collectively referred to as the secretome, which may represent a rich source of novel biologic therapeutics^17–27^. Adult mammary stem cells, as well as luminal, alveolar, and myoepithelial progenitors, have been identified in the mammary gland of mice using lineage tracing^28^. Our lab establishes mammary gland cell cultures enriched for stem and progenitor cells from a wide variety of mammals, including cows^29^, by collecting fresh mammary gland tissues which are enzymatically and mechanically digested to a single-cell solution, and cultured to form mammospheres. Mammospheres are a mixture of stem cells, stem cell progeny, and non-stem progenitor cells^30,31^, which can be expanded to yield cell cultures enriched for these populations. Mammosphere-derived cell cultures are referred to as mammosphere-derived epithelial cells (MDECs)^29^. We previously reported that the secretome of bovine MDECs, delivered as conditioned medium (CM) promotes epithelial cell migration and angiogenesis in vitro^26^. Mass spectrometric analysis of bovine MDEC CM identified the presence of proteins associated with defense and immunity, including known antimicrobial peptides^26^. As has been previously observed, we believe the diversity of the complete bovine stem and progenitor cell secretome leads to broader effects on target cells and tissues than individual secretome components^17,32^.

Recently, a stem cell-like population in bovine milk was identified^33^. This cell population was found to be plastic adherent and capable of differentiation into osteogenic, chondrogenic, and adipogenic lineages, properties used to characterize multipotent mesenchymal stromal cells^34^. Moreover, these cells displayed an immunophenotype consistent with other bovine stem cells^33^. Another contemporary study used single-cell RNA sequencing of cells isolated from bovine milk immediately after collection to identify transcriptomic profiles consistent with immune and epithelial cells^35^. Similar populations have been identified in human breast milk and were shown to be capable of multilineage differentiation and to have therapeutic potential^36–38^.

The goal of the present study was to isolate and characterize cell cultures with stem/progenitor cell characteristics from bovine milk and to evaluate the regenerative and antimicrobial activity of the bovine milk-derived cell (MiDC) secretome. Based on our findings, we propose bovine MiDCs as a source of stem cells and stem cell products that may be used to treat diseases affecting the dairy industry, including mastitis.

## Results

### Bovine milk contains a population of epithelial cells with a stem cell-like phenotype

**Figure 1:**
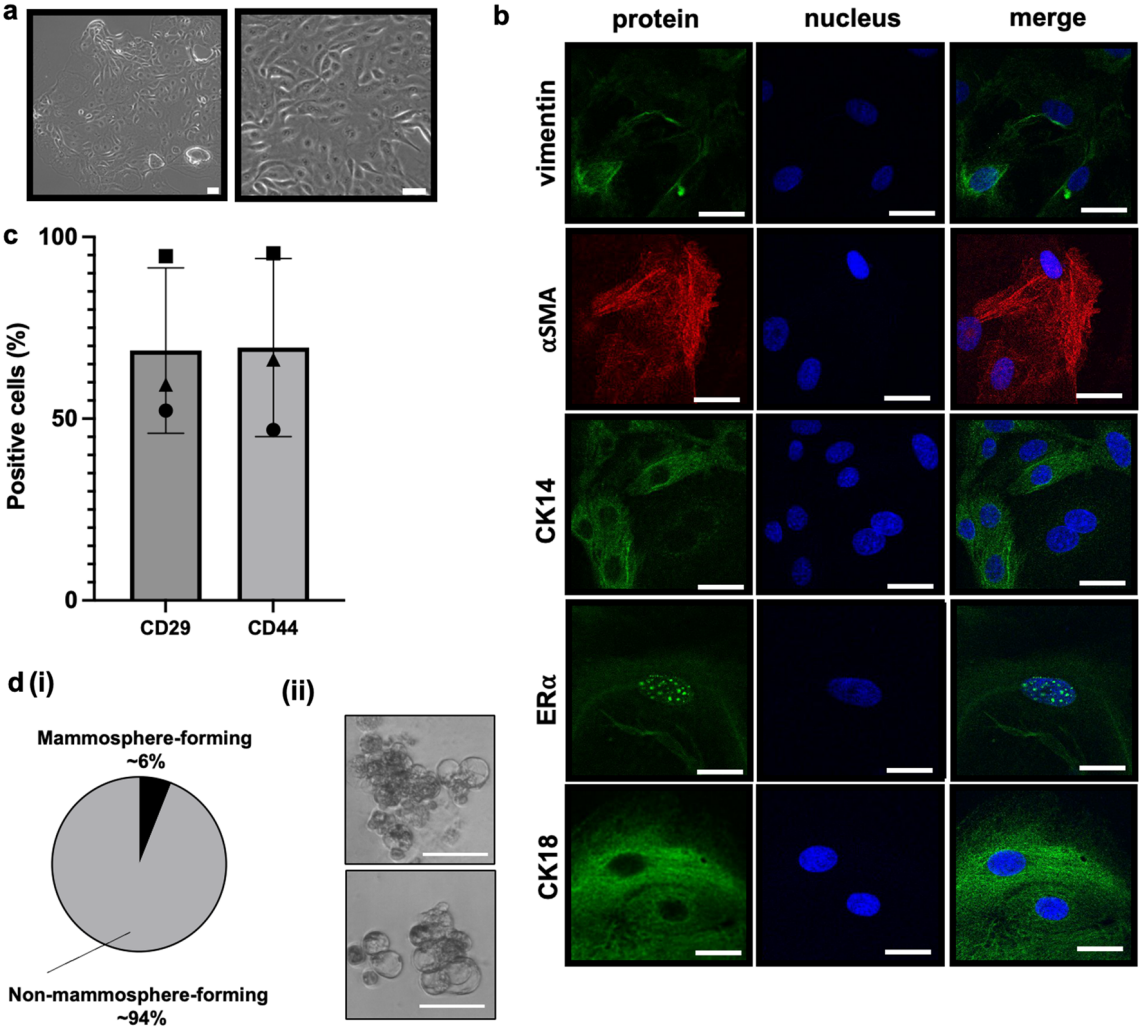
Characterization of milk-derived cells (MiDCs). (**a**) Phase-contrast microscopy images of MiDCs. Scale bars 50 µm. (**b**) Immunofluorescence staining of MiDCs for vimentin, α smooth muscle actin (SMA), cytokeratin (CK) 14, estrogen receptor (ER) α, and CK18. DAPI was used to visualize nuclei. Scale bars 25 µm. (**c**) Flow cytometry analysis of cluster of differentiation (CD) 29 and CD44 expression in MiDCs. Same shapes (i.e., square, circle or triangle) indicate cells from the same cow. (**d**) Pie chart summarizing the percentage of MiDCs that form mammospheres under non-adherent culture conditions (**i**). Representative images of mammospheres from MiDCs. Scale bars 50 µm (**ii**).

Milk-derived cells (MiDCs) were isolated from bovine milk and characterized based on morphology, protein expression, and mammosphere formation. Phase-contrast microscopy images showed that MiDCs are plastic-adherent polygonal cells with cobblestone-like epithelial morphology (Fig. 1a), like bovine mammosphere-derived epithelial cells (MDECs)^26,39^.

Immunofluorescent staining demonstrated that MiDCs are positive for vimentin, α-smooth muscle actin (αSMA), and cytokeratin 14 (CK14), all markers of the basal lineage of mammary epithelial cells^26,40–43^ (Fig. 1b). Some MiDCs express the estrogen receptor α (ERα) and cytokeratin 18 (CK18), two proteins associated with the mammary epithelial luminal lineage^26,43^ (Fig. 1b). Flow cytometric analysis showed that MiDCs exhibit surface marker expression consistent with bovine MDECs^26^, including high levels of CD44 and CD29 (Fig. 1c). Approximately 6% of the MiDC population formed mammospheres when plated under ultra-low adherence conditions (Fig. 1d), close to the ~ 5% mammosphere formation rate observed for bovine MDECs^39^. Since mammospheres are enriched in mammary stem and progenitor cells^30,31^, this indicates that MiDC cultures contain cells with stem/progenitor cell-like properties.

### Single-cell RNA sequencing shows that MiDCs are composed of two distinct cell populations that share a common progenitor

**Figure 2:**
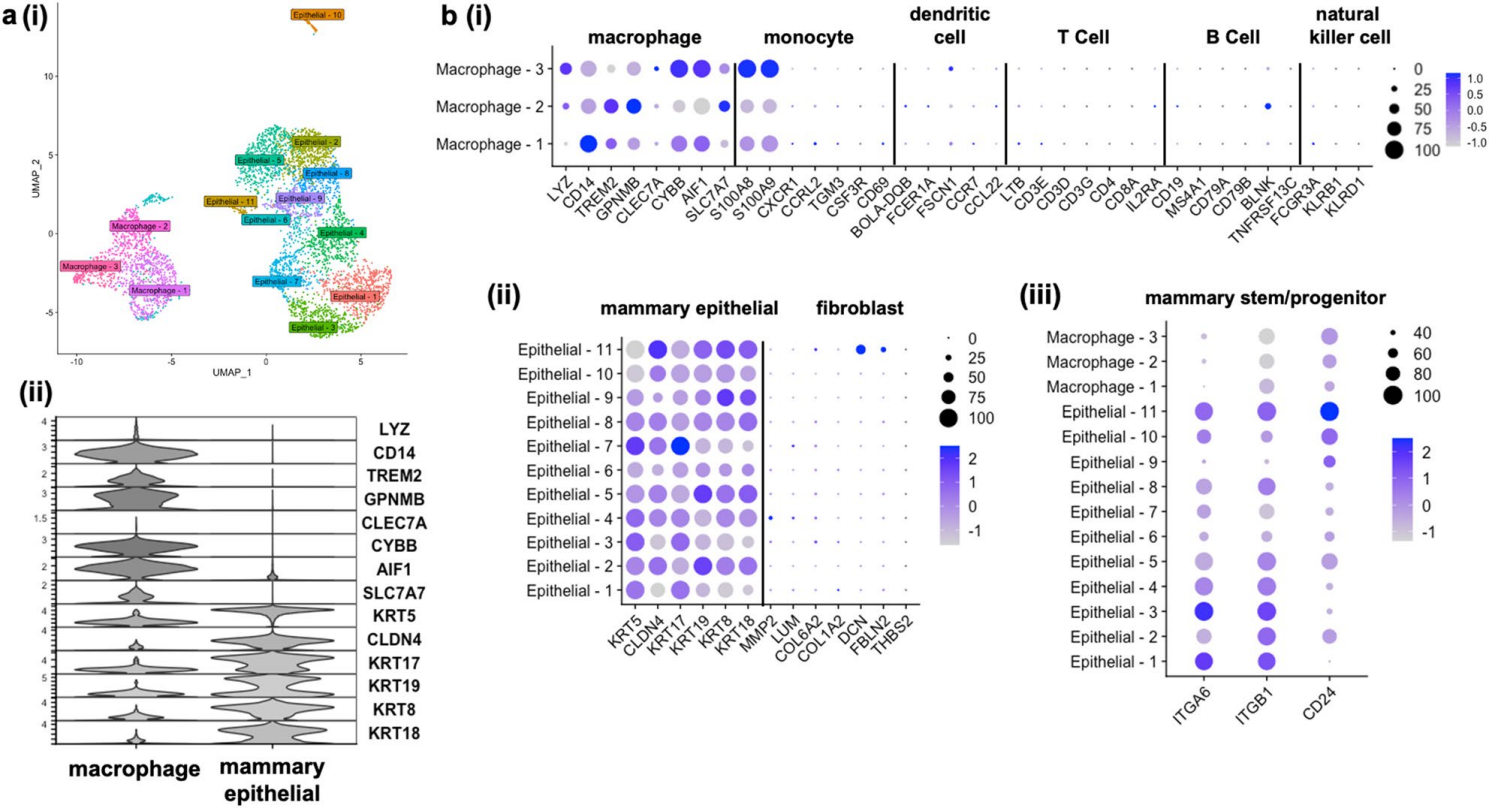
Single cell RNA sequencing (scRNA-seq) of milk-derived cells (MiDCs). (**a**) Uniform manifold approximation and projection plot of scRNA-seq data from MiDCs. Each dot represents one cell (**i**). Stacked violin plot showing the expression of common macrophage and mammary epithelial markers by subpopulation. The Y-axis represents the logarithmically normalized expression level of each gene in the dataset (**ii**). (**b**) Dot plot of macrophage clusters showing expression of known immune cell markers (**i**), of all epithelial clusters showing their expression of known mammary epithelial and fibroblast markers (**ii**), and off all 14 clusters showing their respective expression of known mammary stem and progenitor cell markers (**iii**). Circle size corresponds to percentage of cells expressing each marker (relative size showing percent expressed shown to the right of each plot with numbers representing percentages) and circle color represents the average expression of each marker on a log2 scale (color scheme for relative expression shown to the right of each plot ranging from light gray to dark blue).

Single-cell RNA sequencing (scRNA-seq) was performed to to provide the first unbiased transcriptomic profile of bovine MiDCs. Two distinct subpopulations of cells were identified, spread across 14 clusters (Fig. 2a(i)). Using previously established genetic markers of bovine milk cells^35^, these subpopulations resemble a macrophage lineage and a mammary epithelial lineage (Fig. 2a(ii)). The 3 macrophage clusters express genes associated with monocyte-like cells, such as *S100A8* and *S100A9*, but did not, or only very lowly, express genes associated with dendritic cells, T cells, B cells, and natural killer cells (Fig. 2b(i)).

**Figure 3:**
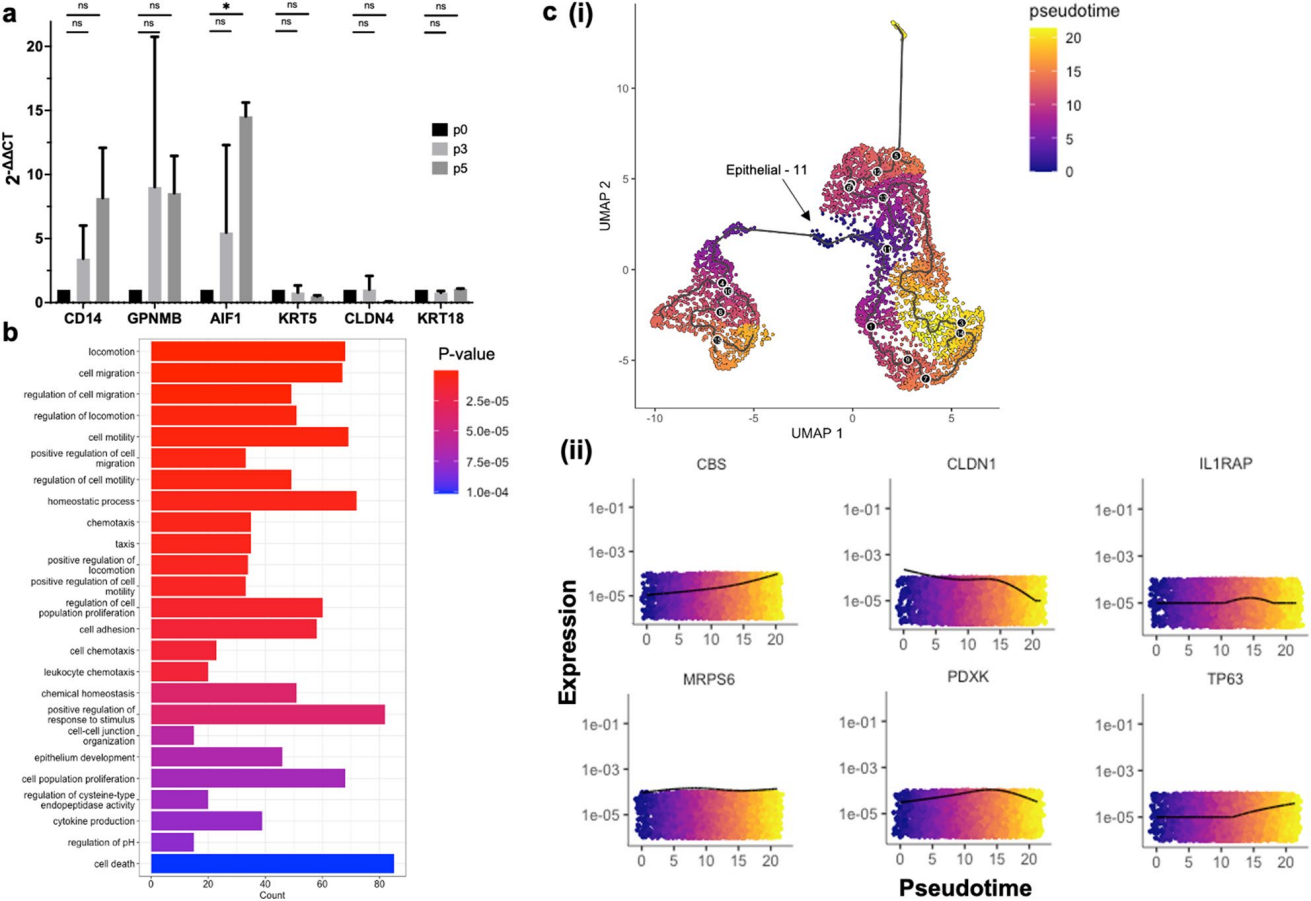
RT-qPCR validation, gene ontology (GO) term enrichment, and pseudotime analysis, of the milk-derived cells (MiDCs) single cell RNA sequencing (scRNA-seq). (**a**) RT-qPCR analysis of *CD14, GPNMB*, *AIF1, KRT5, CLDN4* and *KRT18* expression in MiDCs over 5 passages. n = 3 for passages 3 and 5 and n = 1 for passage 0. The mean is shown with the standard deviation (**b**). GO term enrichment for biological processes based on the top 10 most highly expressed genes in each cluster. Colors represent the adjusted P-score and length of bars represent the count. (**c**) Pseudotime analysis showing a UMAP projection colored by pseudotime, with a putative trajectory (black line) transposed over the plot (**i**) and the top 6 most differentially expressed genes over pseudotime, with colors and x-axis corresponding to pseudotime and y-axis representing expression levels (**ii**). *P < 0.05, *ns* not significant.

The 11 mammary epithelial cell clusters do not express genes associated with fibroblasts, except for cluster “Epithelial-11” that shows a moderate expression of *DCN* (Fig. 2b(ii)). Looking across all 14 clusters, certain clusters that fall within the epithelial lineage group, most notably cluster “Epithelial-11”, show a higher expression of genes associated with mammary stem/progenitor cell markers^30,42^, such as *ITGA6*, *ITGB1*, and *CD24* (Fig. 2b(iii)). To validate these findings, expression of a selection of identified genes that were (1) highly expressed and (2) associated with the macrophage clusters (*CD14, GPNMB* and *AIF1*) and the mammary epithelial cell clusters (*KRT5, CLDN4* and *KRT18*), was assessed by qRT-PCR in MiDCs cultures from multiple cows over multiple passages. Similar gene expression patterns, with largely non-significant differences, apart from *AIF1*, were noted over the multiple passages and across the different MiDC cultures (Fig. 3a), indicating that MiDCs remain stable in culture over multiple subcultures, at least based on expression of selected genes.

We next scanned the scRNA-seq data for genes associated with cell migration, angiogenesis, and defense and immunity, functions of therapeutic value for the treatment of mastitis. Many of the genes correspond to proteins that were previously identified by mass spectrometry in the secretome of bovine mammosphere-derived epithelial cells (MDECs)^26^ (Table 1). We carried out a gene ontology (GO) term enrichment for biological processes based on the top 10 most highly expressed genes in each cluster and found that many of the dominant pathways are related to cell motility (Fig. 3b), suggesting that the secretome of MiDCs may exert functional effects like the secretome of MEDCs^26^. To provide an overview of the developmental trajectories and differentiation potential of MiDCs, we subjected the scRNA-seq data to pseudotime analysis. Pseudotime analysis is a nonlinear manifold learning technique that utilizes machine learning to reconstruct the order of gene expression changes a cell executes to progress from one state to another. Based on the data, cells are placed on a trajectory depicting how far they cell have progressed along a biologic process^44,45^. Analysis of the MiDC data set revealed that the earliest principal node of the putative developmental trajectory lies within cluster “Epithelial-11” (Fig. 3c(i)), which is the cluster expressing the highest level of mammary progenitor markers (Fig. 2b(iii). Further analysis of the pseudotime data identified several variably expressed genes over the pseudotime line, with the top 6 most variably expressed across the trajectory being *CBS*, *CLDN1*, *IL1RAP*, *MRPS6*, *PDXK,* and *TP63* (Fig. 3c(ii)).

**Table 1:** Select transcripts expressed in milk-derived cells (MiDCs).

Gene product	Abbreviation
Genes associated with cell migration	
Hepatocyte growth factor activator*	*HGFAC*
Hepatocyte growth factor receptor*	*MET*
Insulin growth factor binding protein 2*	*IGFBP2*
Insulin growth factor binding protein 3	*IGFBP3*
Insulin growth factor binding protein 4	*IGFBP4*
Insulin growth factor binding protein 5	*IGFBP5*
Insulin growth factor binding protein 6*	*IGFBP6*
Insulin growth factor binding protein 7	*IGFBP7*
Transforming growth factor beta 1*	*TGFB1*
Transforming growth factor beta 2	*TGFB2*
Fibroblast growth factor 1*	*FGF1*
Fibroblast growth factor 2	*FGF2*
Genes associated with angiogenesis	
Vascular endothelial growth factor A*	*VEGFA*
Angiopoietin-related protein 1	*ANGPTL1*
Angiopoietin-related protein 2	*ANGPTL2*
Angiopoietin-related protein 3*	*ANGPTL3*
Angiopoietin-related protein 4	*ANGPTL4*
Genes associated with defense and immunity	
Peptidoglycan recognition protein 1*	*PGLYRP1*
Peptidoglycan recognition protein 2	*PGLYRP2*
Peptidoglycan recognition protein 3	*PGLYRP3*
Peptidoglycan recognition protein 4	*PGLYRP4*
Lactoferrin*	*LTF*
Collectin 11*	*COEC11*
Zinc-alpha-glycoprotein*	*AZGP1*
Protein S100A11*	*S100A11*
Beta-2-microglobulin*	*B2M*
Pantetheinase*	*VNN1*
Cathepsin*	*CTSH*
Alpha-2-microglobulin*	*A2M*

*Indicates corresponding proteins that were previously identified by mass spectrometry in the secretome of bovine mammosphere-derived epithelial cells (MDECs)^26^.

### The MiDC secretome displays both regenerative and antimicrobial properties

**Figure 4:**
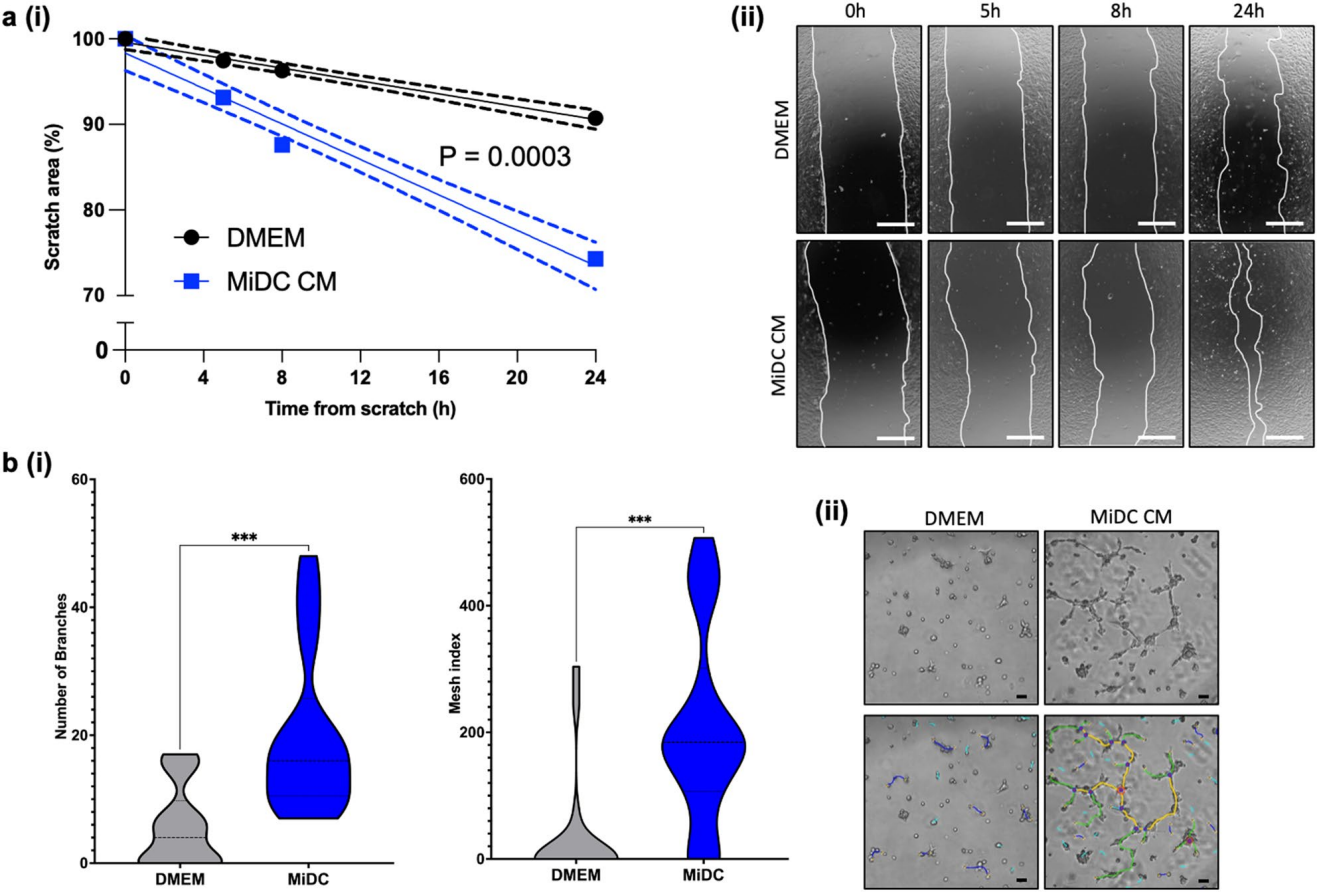
Migration and angiogenesis activity of the milk-derived (MiDC) secretome. (**a**) Measurements of percentage of scratch closure of bovine fibroblasts incubated with DMEM (negative control) or MiDC conditioned medium (CM) after scratching the confluent monolayer (**i**). Representative images at multiple timepoints shown for both conditions. Scale bars 500 µm (**ii**). (**b**) Number of branches and calculated mesh index of bovine endothelial cells (BLMVEC) co-cultured with (negative control) or MiDC MC for 8 h (**i**). Representative images shown for both conditions. Scale bars 100 µm. Branching has been overlayed computationally to indicate structures as follows: red points indicate junctions (i.e., pixels with at least three elements); green represents branches (i.e., elements that have one junction and one extremity); light blue lines represent twigs (i.e., small branches), yellow indicates segments (i.e., elements delimited by two junctions), dark blue indicates isolated elements that are not part of the network (**ii**). P ≤ 0.0002, n = 18.

We previously reported on the regenerative properties of the secretome of bovine MDECs^26^. To assess whether the secretome of MiDCs, collected as conditioned medium (CM), displays similar effects, we performed in vitro migration and angiogenesis assays. For the migration assays, confluent monolayers of mitomycin C-treated bovine mammary fibroblasts were “scratched” to remove cells and incubated with mitomycin C-supplemented MiDC CM or plain DMEM as a control. At each time point tested, fibroblasts incubated with MiDC CM migrated at a significantly faster rate than those incubated with DMEM (Fig. 4a). For angiogenesis assays, the number of branches and the mesh index of structures formed by bovine lung microvascular endothelial cells (BLMVEC) cultured on a basal matrix in the presence of MiDC CM or DMEM were assessed. We found that MiDC CM significantly promoted angiogenesis based on increased branching and a higher mesh index as compared to the DMEM control (Fig. 4b).

**Figure 5:**
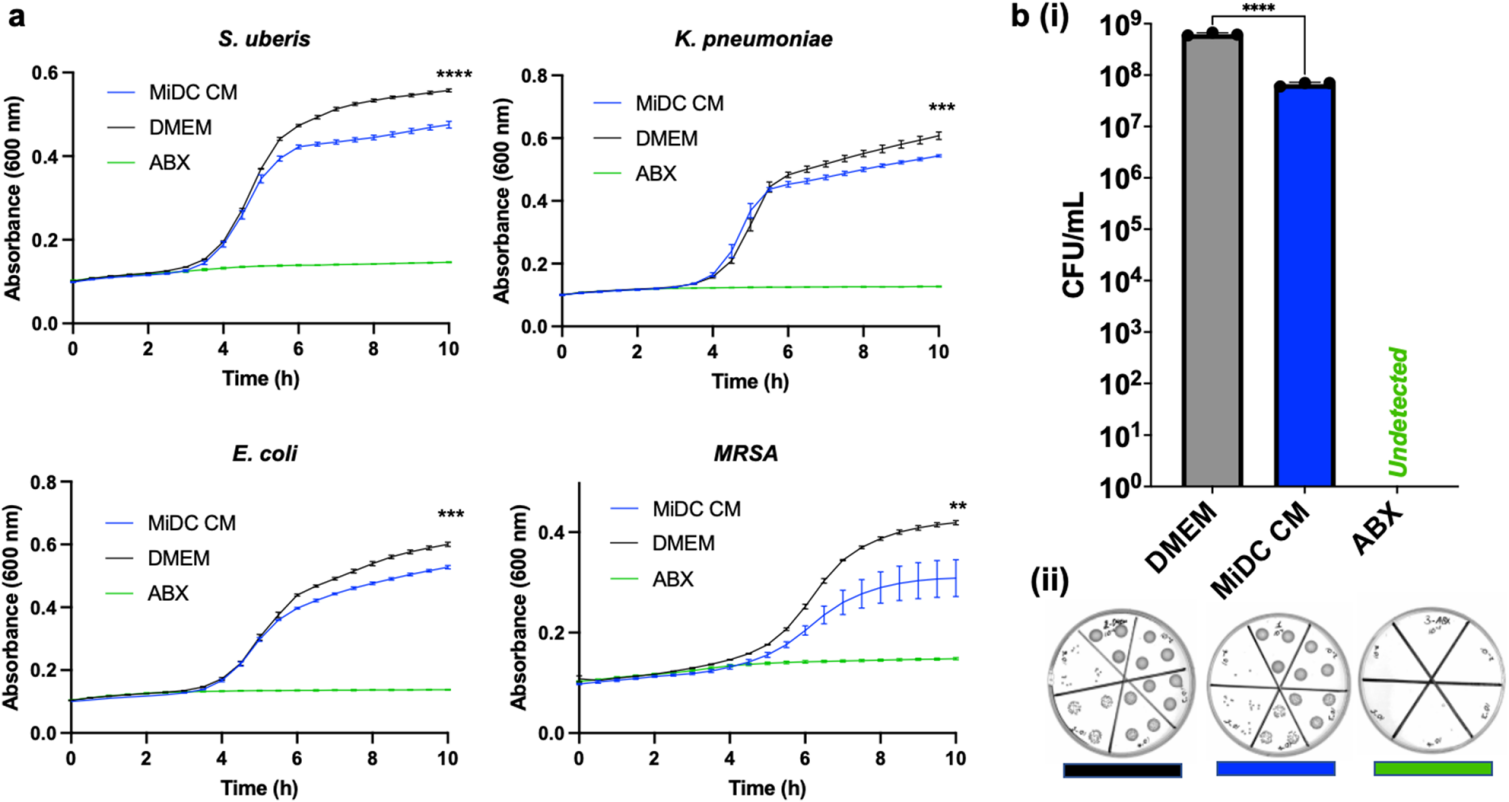
Antimicrobial activity of the milk-derived (MiDC) secretome. (**a**) Ten-hour growth curves of planktonic *Streptococcus* (*S.) uberis*, *Klebsiella (K.) pneumoniae*, *Escherichia (E.) coli,* and *Methicillin-resistant Staphylococcus aureus (MRSA),* incubated with DMEM (negative control), MiDC conditioned medium (CM), or 2× Penicillin/Streptomycin (ABX) (positive control) with absorbance, a proxy for bacterial growth, measured every 30 min at constant 37 °C incubation. Asterisks represent statistical significance between DMEM and MiDC CM at 10 h. Each point represents a timepoint (**b**). Colony-forming units (CFU)/mL of *MRSA* after a 10-h incubation at 37 °C with DMEM (negative control), MiDC conditioned medium (CM), or 2× Penicillin/Streptomycin (ABX) (positive control). Each black dot represents an experimental replicate (**i**). Representative images of the bacterial culture plates are shown for each condition (**ii**). **P < 0.005, ***P < 0.0005, ****P < 0.0001, n = 3.

We previously reported that the secretome of bovine MDECs contains proteins with antimicrobial activity, most notably the antimicrobial peptides lactoferrin, cathelicidin, and peptidoglycan recognition protein^26^. In this study, we evaluated whether the MiDC CM alters the growth of common mastitis-associated pathogens Gram-positive *Streptococcus (S.) uberis,* Gram-negative *Klebsiella (K.) pneumoniae* and Gram-negative *Escherichia (E.) coli*^46^. We also included the multi-drug resistant Gram-positive bacterial strain *Methicillin-resistant Staphylococcus aureus (MRSA)*, which has been documented in mastitis cases^47,48^. Culturing each of these bacterial strains with MiDC CM led to significantly decreased growth over time when compared growth in DMEM (Fig. 5a).

As expected, bacterial growth was fully inhibited when bacteria were grown in DMEM supplemented with a 2× concentrated antibiotic solution (Fig. 5a). These findings were further validated by plating *MRSA*, after 10 h of co-culture for a manual count of colony-forming units (CFU)/mL. The CFU/mL was significantly lower when *MRSA* was co-cultured with MiDC CM as compared to DMEM (Fig. 5b). As expected, CFU/mL was 0 when *MRSA* was grown in DMEM supplemented with a 2× concentrated antibiotic solution (Fig. 5b).

## Discussion

The potential therapeutic use of the stem cell secretome to treat bacterial infections has been reported for animals of agricultural importance such as horses, cows, and chickens^17–19,49,50^. Although udder-derived bovine mammosphere-derived epithelial cells (MDECs), which are enriched for mammary stem and progenitor cells, have been shown to secrete proteins that increase epithelial cell migration, promote angiogenesis, and modulate defense and immune responses^26^, their practical use as a biologic treatment is hindered by the invasive approach needed to isolate these cells from the udder, especially from highly productive lactating cows. Even modern techniques to safely isolate parenchymal biopsies leads to an observable presence of blood in the milk several days post-biopsy, and subcutaneous hematomas for up to a week post collection^51^. Recent studies demonstrated the existence of a stem cell-like population in milk from various mammals, such as humans and cows^33,35,38,52,53^, which could serve as a source of stem cells and the stem cell secretome. The overall aim of this study was to isolate, culture and characterize bovine stem cells from milk and evaluate secretome efficacy in vitro. We successfully isolated and expanded milk-derived cells (MiDCs) with stem and progenitor characteristics and found that the MiDC secretome exhibits both regenerative and antimicrobial activity. These qualities render MiDCs a novel, easily collected, and inexpensive source of stem cells and stem cell-free treatment options for diseases affecting the dairy industry, such as mastitis.

Mastitis is predominantly caused by bacteria, and resulting inflammation can result in severe tissue damage that is irreparable by antibiotics, the most common treatment^54^. We propose that the MiDC secretome can be used as a mastitis treatment as it exerts antimicrobial effects, and promotes tissue repair and regeneration.

The broad-spectrum antimicrobial efficacy of the MiDC secretome against both Gram-positive and Gram-negative bacteria may allow it to target many of the common mastitis-associated pathogens. Coupled with the observed efficacy against antibiotic resistant strains such as *Methicillin-resistant Staphylococcus aureus* (*MRSA*), the MiDC secretome could also provide a novel biologic for cases of bacterial mastitis that persist through multiple different antibiotic treatments. This is particularly relevant as studies show that treatment of food-producing animals with current antibiotics is correlated with an increase in antimicrobial resistance in both humans and animals^9,10^.

We found that the MiDC secretome promotes angiogenesis in vitro which may be therapeutically valuable since (1) clinical cases of mastitis can result in ischemic necrosis, a condition caused by reduced blood flow to the tissue^55^ and (2) ischemic necrosis can be alleviated by neovascularization, as shown in initial clinical studies with patients suffering from peripheral artery disease^56,57^.

Our single cell RNA sequencing (scRNA-seq) of MiDCs revealed 14 different cell clusters based on their transcriptomic profiles and these clusters grouped into two distinct subpopulations of cells, one exhibiting markers of mammary epithelial cells, the other markers of macrophages. The latter is the most common subpopulation of somatic cells found in healthy bovine milk^58^ and has been shown to be present in bovine milk cell populations canonically identified as epithelial^59^. Second, pseudotime analysis of the different cell clusters revealed a common progenitor located in the cluster that expressed the highest level of mammary progenitor markers. Tumor protein p63 (*TP63*) was among the top 6 differentially expressed genes over pseudotime. *TP63* is a transcription factor that serves as a master regulator of mammary cell stemness in mouse models^60,61^, making it consistent with our findings that its expression level may be a proxy for developmental time. This information can be useful to further define a panel of specific markers that can be used to identify the extent of developmental potency in bovine mammary epithelial stem and/or progenitor cells.

We propose bovine MiDCs as an alternative source of mammary stem/progenitor cells that that can be non-invasively and cost-effectively collected from milk and expanded in culture. The functional effects of the MiDC secretome on target cells and tissues can be further explored to determine its potential as a biologic therapeutic for economically important and prevalent udder diseases, such as mastitis.

## Methods

### Cell isolation and culture

Using an automated milking system (DeLaval, Tumba, Sweden), milk was collected, under Cornell University IACUC approval #2012-0101, from four healthy Holstein–Friesian cows that had more than 1 lactation cycle and no clinical signs of mastitis. Each cow was between 2 (lactation 1) and 10 (lactation 8) years old, with an average age of 5 years (lactation 3); their average weight was 726 kg. Cows were confirmed by a licensed veterinarian to be clinically healthy, without any clinical or subclinical mastitis based on their somatic cell count (SCC), which was ≤ 200,000 cells/mL. About 980 mL of milk was combined with 20 mL of 100× antibiotic antimycotic solution (AB/AM) (Corning, Corning, NY) and divided into four 250 mL aliquots. Aliquots were centrifuged at 500×*g* for 10 min (min) at 4 °C, supernatants discarded, and cell pellets were resuspended in 25 mL Hank’s Balanced Salt Solution (HBSS) (Gibco, Waltham, MA), supplemented with 2% AB/AM (HBSS + AB/AM). After adding an additional 25 mL HBSS + AB/AM, cell suspensions were centrifuged at 500×*g* for 5 min at 4 °C, resuspended, and washed again via centrifugation. Cell pellets were then resuspended in 50 mL HBSS + AB/AM, supplemented with 2% fetal bovine serum (FBS) (R&D Systems, Flowery Branch, GA) and centrifuged at 300×*g* for 5 min at room temperature (RT). This was repeated until supernatants were clear. Cells were plated in 6-well culture plates at a concentration of 5 × 10^6^ cells/well in Dulbecco’s Modified Eagle Medium (DMEM)/F12 (50/50) (Corning), supplemented with 10% FBS, 2% B27 (Invitrogen, Waltham, MA), 10 ng/mL basic-fibroblast growth factor (bFGF) (BioVision, Milpitas, CA), 10 ng/mL epidermal growth factor (EGF) (Sigma, Darmstadt, Germany) and 2% AB/AM (EpSC medium). After 2 hours (h) of incubation at 37 °C, medium was aspirated and fresh EpSC medium was added to the wells. Plates were incubated overnight, washed twice with phosphate-buffered saline (PBS) (Corning), and further incubated with daily medium changes until colonies started to appear, around 8–11 days post plating. Colonies were expanded and maintained in EpSC medium, but with 1% Penicillin/Streptomycin (P/S) (Corning) instead of 2% AB/AM. Three cell lines were isolated from three different animals, whereas the fourth was discarded due to a fungal contamination shortly after isolation.

### Immunofluorescence

Upon 70% confluency, cells were detached from culture wells, and 1000 cells were plated on 12 mm glass coverslips and left to grow overnight. Cells were fixed using 4% paraformaldehyde (PFA) (Thermo Scientific, Waltham, MA), and those to be labeled with intracellular antibodies were permeabilized using 0.1% Triton in PBS. Coverslips were incubated with primary antibodies overnight at 4 °C, washed three times with PBS for 2 min at RT, and incubated with fluorescently labeled secondary antibodies for 1 h at RT. After washing three times with PBS for 2 min at RT, 4′,6-diamidino-2-phenylindole (DAPI) (Thermo Scientific) was added for 5 min at RT, and coverslips were washed with PBS. Coverslips were attached to glass slides using aqueous mounting medium (Dako, Santa Clara, CA) and cells were visualized with a confocal laser scanning microscope (Zeiss, Oberkochen, Germany). Staining with isotype control antibodies, diluted at the same dilution, or no primary antibodies was included as a negative control. Antibodies and dilutions are listed in Table 2.

**Table 2:** Antibodies used for immunophenotyping.

Target	Abbreviation	Host	Clone	Dilution	Manufacturer
Vimentin	Vim	Mouse	RV202	1:100	Abcam
Alpha smooth muscle actin	αSMA	Mouse	1A4 (conjugated to AF594)	1:100	Abcam
Cytokeratin 14	CK14	Mouse	LL002	1:100	Abcam
Estrogen receptor alpha	ERα	Mouse	T111.5D11	1:100	Abcam
Cytokeratin 18	CK18	Mouse	C-04	1:100	Abcam
Cluster of differentiation 29	CD29	Mouse	TDM29	1:20	Chemicon
Cluster of differentiation 44	CD44	Rat	IM7	1:20	BD
Mouse IgG (H + L)		Goat	N/A	1:100-IF 1:5-FC	Jackson ImmunoResearch
Rat IgG (H + L)		Goat	N/A	1:20	Sigma

*N/A* not applicable, *IF* immunofluorescence, *FC* flow cytometry.

### Flow cytometry

Cells were resuspended in PBS with 1% Bovine Serum Albumin (BSA) (Thermo Scientific) and incubated with primary antibodies for 1 h on ice. Cells were then washed with PBS + 1% BSA by centrifugation at 300×*g* for 7 min at 4 °C and resuspended in PBS + 1% BSA with secondary antibodies for 20 min on ice. Cells were washed again, as described above, resuspended in PBS + 1% BSA, and analyzed on a LSRFortessa flow cytometer (BD, Franklin Lakes, NJ) operated by FACSDiva (BD) software. Additional data processing and analysis was conducted using FlowJo (BD). Staining with isotype control antibodies or no primary antibodies was included as negative control. Antibodies and dilutions are listed in Table 2.

### Mammosphere formation assays

Mammosphere formation assays were conducted as previously described^39,62^. Briefly, cells were cultured at concentrations of 2, 5, 10, 25, 50 and 100 cells per well in 8 wells of an ultra-low adherence 96-well plate (Corning). After 10 days, each well was assessed for the presence of mammospheres (defined as cell structures greater than 40 μM in diameter^39,63^) and results were put into the Extreme Limiting Dilution Analysis (ELDA) software (https://bioinf.wehi.edu.au/software/elda/) to calculate mammosphere forming cell frequency.

### Single-cell RNA sequencing (scRNA-seq) and analysis

Cells were prepared using the 10× Genomics Single Cell platform (10× Genomics, Pleasanton, CA), following manufacturer guidelines. Briefly, cells were collected in suspension and diluted to a concentration of 8 × 10^5^ cells/mL in PBS. Cell viability of 80% or greater was confirmed using a Countess 3 FL automated cell counter (Invitrogen). The scRNA-seq library was generated using the Chromium Next GEM Single-cell 3′ v3.1 assay (10× Genomics), according to manufacturer protocols. The cDNA library was assessed for quality and concentration using a Fragment Analyzer System (Agilent Technologies, Santa Clara, CA). Sequencing parameters recommended by 10× Genomics were used on a NextSeq 2000 (Illumina, San Diego, CA) using the NextSeq 2K P3 100 bp kit (Illumina).

Sequencing reads were aligned and assessed for quality using the Cell Ranger v6.1.1^64^ alignment software (10× Genomics). Raw base call files from the NextSeq 2000 (Illumina) sequencer were converted to FASTQ using the ‘mkfastq’ command. The FASTQ file was aligned to the *Bos taurus* ARS-UCD1.2^65^ genome with Ensembl 106 annotation release using the ‘count’ function of Cell Ranger.

The R (version 4.2.2) environment was used for all data analysis using the Seurat^66^ (version 4.3.0) and monocle3^45^ (version 1.3.1) packages. Quality control (QC) filtering was conducted by removing cells with more than 8% mitochondrial genes, fewer than 1000 UMI counts, or fewer than 700 features. Prior to filtering, there were 5577 cells with 97.8% of reads mapped to the genome, 36,488 mean reads per cell, 3050 median genes per cell and 16,455 total genes detected. Following QC filtering, 4877 cells remained. Normalization was done using the ‘NormalizeData’ function of Seurat with a normalization method of ‘LogNormalize’ and a scale factor of 10,000. Cells were scored for cell cycle using the ‘CellCycleScoring’ function and human genes were converted to their bovine equivalents using ‘biomaRt’^67^. Data was scaled and potential sources of unspecific variation were regressed out using the ‘ScaleData’ function and regressing the mitochondrial gene proportion, the cell cycle effect and UMI count. ‘FindVariableFeatures’ was used to identify 2000 variable genes and principal component (PC) analysis was used to plot the variability between cells. Specifically, the first 10 PCs were used to generate a two-dimensional diagram by means of Uniform Manifold Approximation and Projection (UMAP). Subpopulations of cells were clustered using the ‘FindClusters’ function, using a resolution of 0.8. In order to identify markers and name clusters, the function ‘FindAllMarkers’ was used, and cell types were manually assigned by utilizing previously described markers^35^. Pseudotime analysis was conducted following the monocle3 vignette, and the earliest principal node was obtained using the ‘get_earlist_principal_node’ function. Gene ontology (GO) enrichment for biological processes was done by selecting the markers identified in ‘FindAllMarkers’ and executing the ‘enrichGO’ function of ‘clusterProfiler’ (Version 3.0.4)^68^ on that dataset using the ‘org.Bt.eg.db’ database.

### Quantitative reverse-transcription PCR (qRT-PCR)

RNA was extracted using the RNeasy Mini Plus kit (Qiagen, Hilden, Germany) and cDNA was synthesized using the iScript gDNA Clear cDNA synthesis kit (BioRad), as per manufacturer’s protocols. Primers were designed using Primer3^69^, using sequence data from NCBI GenBank. In addition to removing gDNA using the RNeasy and iScript kits, primers were designed to span intronic regions, when possible, to avoid gDNA amplification (Table 3). qPCR was performed using PowerTrack SYBR Green MasterMix (Applied Biosystems, Waltham, MA) and *GAPDH* was included as reference gene. Amplification was performed on a QuantStudio3 Thermal Cycler (Applied Biosystems).

**Table 3:** qPCR primer sequences.

Gene product	Abbreviation	Forward primer (5′–3′)	Reverse primer (5′–3′)
Cluster of differentiation 14	*CD14*	TGATCTCAGCTGCAACAAGC	AGGGATTTCCGTCCAGAGTC
Transmembrane glycoprotein NMB	*GPNMB*	GTGAGGAGAGCCTTCAGTGG	GTGAGGAGAGCCTTCAGTGG
Keratin 5	*KRT5*	TGCTGAGGGAGTACCAGGAG	TGCTGAGGGAGTACCAGGAG
Claudin 4	*CLDN4*	CACTCTGCCAGCATTCTCTG	AAACCTGTCCGTCCACTCTG
Keratin 18	*KRT18*	GCAGACCGCTGAGATAGGAG	CAAGCTGGCCTTCAGATTTC
Glyceraldehyde 3-phosphate dehydrogenase	*GAPDH*	CACAGTCAAGGCAGAGAACG	TACTCAGCACCAGCATCACC

### Collection of conditioned medium

Conditioned medium (CM) was collected, as described previously^18,19,23,26^, from cell cultures isolated from three different cows. Briefly, cells were grown for at least two passages in antibiotic-free EpSC medium and upon confluency, plated at a concentration of 1 × 10^6^ in a T75 flask and allowed to grow overnight in antibiotic-free EpSC medium. Cell monolayers were washed twice with PBS and cultured in 6 mL DMEM for 24 h. Medium was collected and centrifuged at 300×*g* for 10 min at RT, decanted into a new tube, and centrifuged again to remove any cellular debris.

### Scratch assays

Scratch assays were carried out, as previously described^22^ with some modifications. Bovine mammary fibroblasts, isolated from the mammary gland tissue of a cow sacrificed for an unrelated study, were seeded in a 6-well plate. Upon 100% confluency, the proliferation inhibitor mitomycin C was added at concentration of 20 µg/mL. Using a P1000 pipette tip, a single scratch was made across the middle of the well. Fibroblasts were washed with PBS and cultured with MiDC CM or DMEM + 1% FBS as negative control, both supplemented with mitomycin C. At time points 0, 5, 8, and 24 h after scratching, images of the scratch were taken at the same two locations of each well. Assays were performed in triplicate and scratched areas without cells were calculated using ImageJ and averaged.

### Angiogenesis assays

Endothelial tube-like formation was assessed, as previously described^21^, using an angiogenesis kit (Abcam) and following manufacturer’s instructions. Briefly, bovine lung microvascular endothelial cells (BLMVEC) (kind gift from Dr. Theresa Curtis, State University of New York at Cortland) were plated on wells coated with extracellular matrix, provided in the kit, in a 96-well plate and incubated with DMEM + 1% FBS (negative control), MiDC CM, or DMEM + 1% FBS with 40 µM suramin, an inhibitor of angiogenesis, for 8 h at 37 °C. BLMVEC were washed, stained with an FITC fluorescent dye, provided in the kit, and imaged using a ZOE fluorescent imager (BioRad) and phase-ring microscope (Olympus, Tokyo, Japan). Three phase-ring images from each well were analyzed using the Angiogenesis Analyzer plug-in^70^ for ImageJ. Each test condition was run in duplicate wells.

### Antimicrobial assays and CFU counts

Antimicrobial assays were conducted, as previously described^26^ with the following modifications. Briefly, four bacterial strains (Supplemental Table 1) were plated on Luria–Bertani (LB) agar and allowed to form colonies overnight at 37 °C. A single colony of each strain was expanded into LB broth and grown overnight at 37 °C on a shaker. Bacteria were diluted to a concentration of 100 colony forming units (CFU)/µL. Consequently, 750 µL of CM was combined with 250 µL of bacteria and plated into four wells of a 96-well plate at 200 µL per well. Four technical replicates of DMEM and DMEM supplemented with 2× P/S were included as negative and positive controls, respectively. Plates were covered and incubated for 10 h at 37 °C in a Tecan Infinite 200 Pro plate reader (Tecan, Mannedorf, Switzerland). Absorbance was measured at 600 nm every 30 min. Data were plotted, and analyzed using Prism 9 (GraphPad, San Diego, CA).

To count CFUs, four technical replicates of the antimicrobial assay were combined after completion of the incubation and absorbance measurement from each experimental condition (MiDC CM, DMEM and Antibiotic). Serial dilutions were performed, by diluting the bacterial co-cultures in LB broth in increments of 10^–1^, from 10^–1^ to 10^–^6. Three 10 µL aliquots of each dilution between 10^–1^ and 10^–6^ were plated for each condition on LB agar plates, inverted and incubated at 37 °C for 18–20 h. Plates were imaged using a ChemiDoc MP imager (BioRad). Colonies were counted from images manually at the highest dilution where single colonies could be identified.

### Statistical analysis

Statistical analysis for all functional experiments was performed used Prism 9 (GraphPad). For the qPCR data, a two-way ordinary analysis of variance (ANOVA) was performed. For the angiogenesis assays, an unpaired t-test of DMEM versus MiDC CM was performed for both branches and mesh index metrics. P values were two-tailed for both assays. Statistics for the scratch assay were obtained by performing a two-way repeated measure ANOVA. Sphericity was not assumed and an alpha of 0.05 was selected. P value was evaluated for the source of variation. A simple linear regression was performed on the points for each biogroup to define a slope, x-intercept, and y-intercept, respectively. 95% confidence intervals for each condition were calculated.

### Supplementary Information

Supplementary Table 1.

## Data Availability

The original sequencing data discussed in this publication have been deposited in NCBI’s Gene Expression Omnibus^71^ and are accessible through GEO Series accession number GSE229898 (https://www.ncbi.nlm.nih.gov/geo/query/acc.cgi?acc=GSE229898).
