# Supplementary material for: Bovine milk-derived cells express transcriptome markers of pluripotency and secrete bioactive factors with regenerative and antimicrobial activity
Source: Sci Rep. 2023 Aug 3;13:12600. doi: 10.1038/s41598-023-39833-9 (PMC10400535; doi:10.1038/s41598-023-39833-9)
Supplement: Supplementary file 1 — Supplementary Table 1. [file 41598_2023_39833_MOESM1_ESM.docx]

**Supplemental Table 1. Bacterial strains used in the study.**

| Species | Isolate type | Isolation species/site |
| --- | --- | --- |
| *Streptococcus uberis* | Mastitis culture | *Bos taurus*, left hind quarter |
| *Klebsiella pneumoniae* | Mastitis culture | *Bos taurus*, left front quarter |
| *Escherichia coli* | Mastitis culture | *Bos taurus*, right front quarter |
| *Methicillin-resistant Staphylococcus aureus* subsp. a*ureus* Rosenbach | BAA-1556 (ATCC) | *Homo sapiens*, wrist abscess |

Mastitis culture: indicates that the bacteria are field isolates collected from a mastitic cow without genotyping. ATCC: American Type Culture Collection.
